# Impact of metformin on cardiovascular disease: a meta-analysis of randomised trials among people with type 2 diabetes

**DOI:** 10.1007/s00125-017-4337-9

**Published:** 2017-08-02

**Authors:** Simon J. Griffin, James K. Leaver, Greg J. Irving

**Affiliations:** 1 The Primary Care Unit, Institute of Public Health, School of Clinical Medicine, University of Cambridge, Box 113 Cambridge Biomedical Campus, Cambridge, CB2 0SR UK; 2 MRC Epidemiology Unit, Institute of Metabolic Science, School of Clinical Medicine, University of Cambridge, Cambridge Biomedical Campus, Cambridge, UK

**Keywords:** Cardiovascular disease, Meta-analysis, Metformin, Review, Systematic review

## Abstract

**Aims/hypothesis:**

Metformin is the most-prescribed oral medication to lower blood glucose worldwide. Yet previous systematic reviews have raised doubts about its effectiveness in reducing risk of cardiovascular disease, the most costly complication of type 2 diabetes. We aimed to systematically identify and pool randomised trials reporting cardiovascular outcomes in which the effect of metformin was ‘isolated’ through comparison to diet, lifestyle or placebo.

**Methods:**

We performed an electronic literature search of MEDLINE, EMBASE and the Cochrane Library. We also manually screened the reference lists of previous meta-analyses of trials of metformin identified through a MEDLINE search. We included randomised controlled trials of adults with type 2 diabetes comparing any dose and preparation of oral metformin with no intervention, placebo or a lifestyle intervention and reporting mortality or a cardiovascular outcome.

**Results:**

We included ten articles reporting 13 trials (including a total of 2079 individuals with type 2 diabetes allocated to metformin and a similar number to comparison groups) of which only four compared metformin with placebo and collected data on cardiovascular outcomes. Participants were mainly white, aged ≤65 years, overweight/obese and with poor glycaemic control. Summary estimates were based on a small number of events: 416 myocardial infarctions/ischaemic heart disease events in seven studies and 111 strokes in four studies. The UK Prospective Diabetes Study (UKPDS) contributed the majority of data to the summary estimates, with weights ranging from 52.3% for myocardial infarction to 70.5% for stroke. All outcomes, with the exception of stroke, favoured metformin, with limited heterogeneity between studies, but none achieved statistical significance. Effect sizes (Mantel–Haenszel RR) were: all-cause mortality 0.96 (95% CI 0.84, 1.09); cardiovascular death 0.97 (95% CI 0.80, 1.16); myocardial infarction 0.89 (95% CI 0.75, 1.06); stroke 1.04 (95% CI 0.73, 1.48); and peripheral vascular disease 0.81 (95% CI 0.50, 1.31).

**Conclusions/interpretation:**

There remains uncertainty about whether metformin reduces risk of cardiovascular disease among patients with type 2 diabetes, for whom it is the recommended first-line drug. Although this is mainly due to absence of evidence, it is unlikely that a definitive placebo-controlled cardiovascular endpoint trial among people with diabetes will be forthcoming. Alternative approaches to reduce the uncertainty include the use of electronic health records in long-term pragmatic evaluations, inclusion of metformin in factorial trials, publication of cardiovascular outcome data from adverse event reporting in trials of metformin and a cardiovascular endpoint trial of metformin among people without diabetes.

**Electronic supplementary material:**

The online version of this article (doi:10.1007/s00125-017-4337-9) contains a slideset of the figures for download, which is available to authorised users.

## Introduction

The aims of prescribing medication to lower glucose among people with type 2 diabetes are to reduce the symptoms of hyperglycaemia and the risk of microvascular and macrovascular complications. Since ancient times, a range of therapies have been effective at alleviating the polydipsia and polyuria associated with raised blood glucose levels. Twenty years ago, trial evidence finally emerged showing that lowering blood glucose reduced risks of microvascular complications among people with type 2 diabetes [1]. However, subsequent randomised trials evaluating the effects of intensive treatment for the regulation of blood glucose have highlighted concerns about adverse effects, in particular hypoglycaemia and mortality, and demonstrated inconsistent findings for risk of micro- and macrovascular complications [2, 3]. The evidence of benefit appears stronger for micro- than macrovascular disease [4], albeit the latter represents the greatest burden to healthcare and society. The situation is further complicated by the heterogeneity in findings of observational studies [5] and randomised trials of different drugs (and combinations of drugs [6]) used to lower blood glucose, even those with apparently similar pharmacological targets [7–10].

The biguanide metformin has had an interesting history. After an inauspicious debut, a somewhat circuitous route (including use as a treatment for infectious disease), the withdrawal of phenformin and buformin (the only other biguanides used to lower blood glucose) following trial evidence of harm [11] and publication of the UK Prospective Diabetes Study (UKPDS) results, showing evidence of cardiovascular benefits [12], metformin was introduced in the USA in 1995 and has emerged as the first-choice and most-prescribed oral medication to lower blood glucose worldwide [13]. However, recent systematic reviews have raised doubts about the effectiveness of metformin in reducing risk of complications [14, 15]. In these reviews, data were pooled from predominantly small trials with short follow-up in which metformin was evaluated against a range of comparators that have heterogenous associations with risk of cardiovascular disease. This has constrained interpretation of the benefits and harms of metformin. Since 2008, the US Food and Drug Administration (FDA) have required demonstration of cardiovascular safety prior to the licensing of new glucose-lowering drugs. Several placebo-controlled trials of glucose-lowering drugs, incorporating cardiovascular endpoints, have reported findings or are underway [16]. Consequently, the availability of data to inform treatment guidelines and prescribing decisions for patients is increasing. In this changing context, it therefore seems timely to review the evidence for cardiovascular disease prevention with metformin.

As part of a series of papers to acknowledge the sixtieth anniversary of the first use of metformin for the treatment of type 2 diabetes in this issue of *Diabetologia*, we appraise the evidence concerning the effectiveness of metformin in preventing cardiovascular disease in patients with type 2 diabetes by undertaking a meta-analysis. We aimed to systematically identify and pool randomised trials reporting cardiovascular outcomes in which the effect of metformin was ‘isolated’ through comparison to diet, lifestyle or placebo, rather than alternative glucose-lowering medication.

## Methods

We undertook a systematic review and meta-analysis following the Preferred Reporting Items for Systematic Reviews and Meta-Analyses (PRISMA) guidelines [17].

### Search strategy

We performed an electronic literature search of MEDLINE (1 January 1967 to 6 February 2017; www.ncbi.nlm.nih.gov/m/pubmed), EMBASE (1 January 1947 to 28 February 2017; https://login.webofknowledge.com) and the Cochrane Library (1 January 1967 to 28 February 2017; www.cochranelibrary.com;), with no language limits, using search terms (shown in the Text box) adapted from an earlier Cochrane review [18]. We also performed an electronic literature search of MEDLINE, with no language or date limits, for papers with ‘meta-analysis’ and ‘metformin’ in the title. We manually screened the reference lists of identified meta-analyses. Finally, we manually screened the reference list of a recent systematic review of cardiovascular endpoint trials of glucose-lowering medication [16].
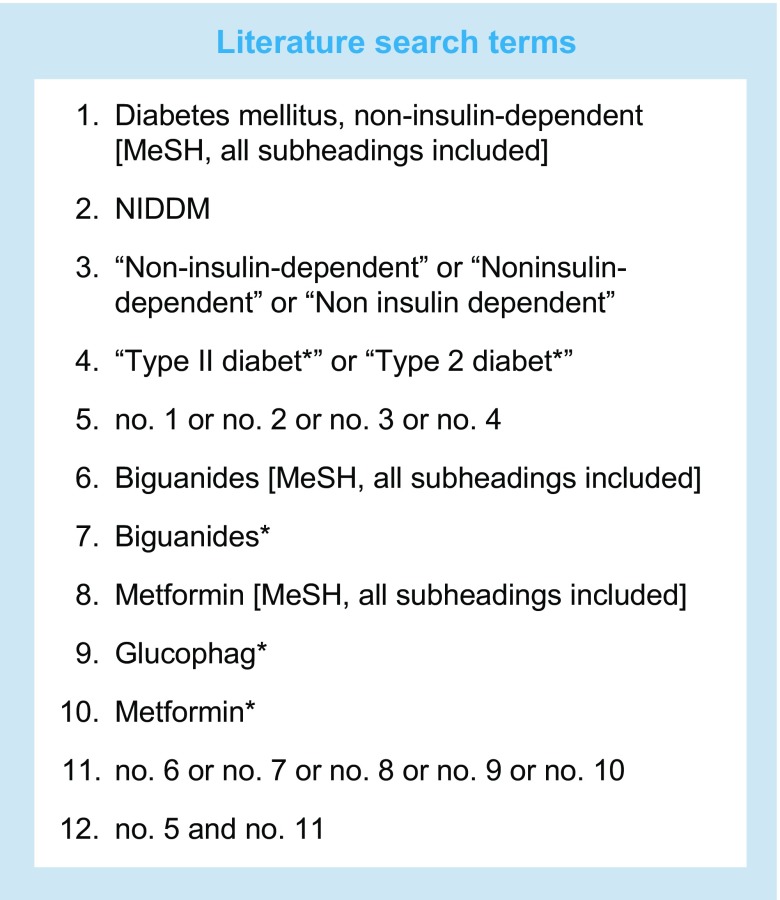


### Study selection

We included studies if they fulfilled all of the following criteria: randomised controlled trial among adults with type 2 diabetes comparing any dose and preparation of oral metformin with no intervention, or with placebo or a lifestyle intervention, and reporting mortality or a cardiovascular outcome (cardiovascular death, myocardial infarction, stroke or peripheral vascular disease) as a primary or secondary outcome or adverse event. There was no restriction based on duration of follow-up. We included studies in which metformin was combined with another drug as long as the comparator group was given the other drug at the same dose as used in the metformin combined therapy group, thereby controlling for the effects (either positive or negative) of the other drug and ‘isolating’ the impact of metformin. We excluded quasi-experimental studies, crossover and observational studies, and studies including children, pregnant women and people with impaired glucose tolerance. Two authors (G. Irving and J. Leaver) independently screened titles and abstracts identified by the MEDLINE search to exclude papers that were clearly not relevant. Any disagreement was solved by discussion with S. Griffin. The full text was examined by one author (G. Irving) if a definite decision to reject could not be made based on title and abstract alone. The full text of all included studies was reviewed by a second author (S. Griffin). After excluding duplicates, one author (G. Irving) repeated the process for the results of the EMBASE search.

### Data extraction and synthesis

Data concerning study size, interventions, inclusion criteria, duration of follow-up, participant characteristics (for the metformin group) and outcomes were extracted independently by at least two out of the three authors and any disagreement was resolved by discussion. We assessed risk of bias in included trials according to the methods recommended by the Cochrane Collaboration. We combined outcome data from different studies using fixed effects meta-analysis and presented data as Mantel–Haenszel (M–H) RRs and 95% CI. When one article reported multiple comparisons, we treated each as a separate study (these are labelled as ‘a’ and ‘b’ in tables and figures). For factorial designs, we included overall comparisons between metformin and placebo groups. When data from one study were reported in more than one article we extracted the most recently published data. For outcomes with data from three or more studies, we assessed heterogeneity between studies using the *I* ^2^ statistic. We assessed the risk of publication bias by producing a funnel plot for all-cause mortality. We used RevMan version 5.3 for analyses (http://community.cochrane.org/tools/review-production-tools/revman-5/revman-5-download).

## Results

**Fig. 1:**
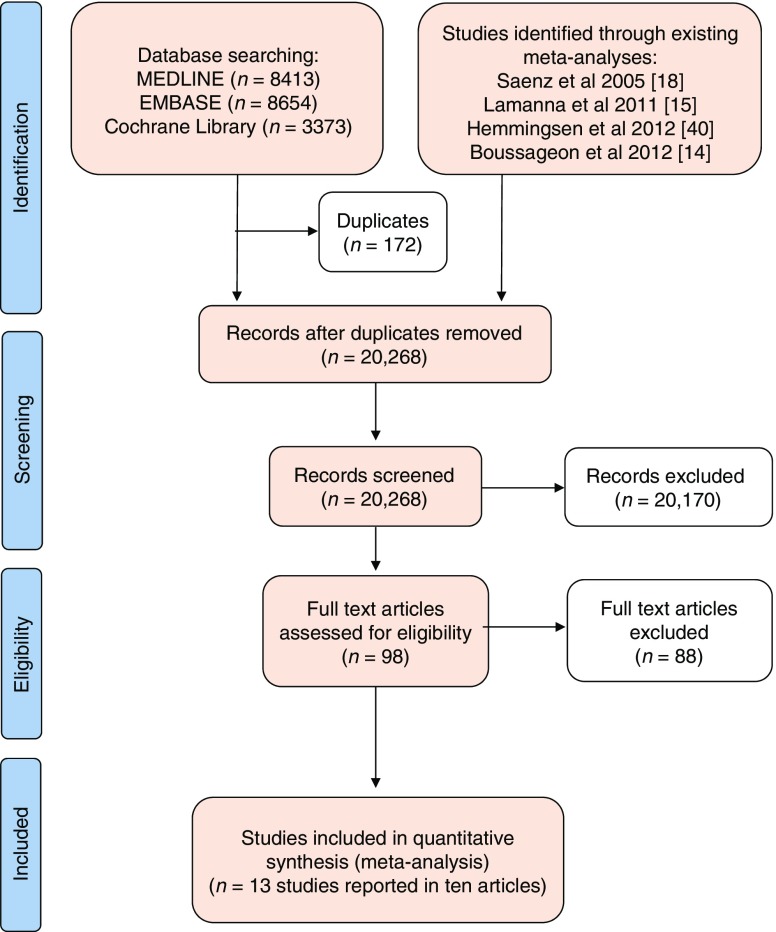
Preferred Reporting Items for Systematic Reviews and Meta-Analyses (PRISMA) flow diagram [40]

After removal of duplicates, the initial electronic search identified 20,268 articles (Fig. 1). Following screening of titles and abstracts, we reviewed the full text of 98 articles and included ten articles reporting 13 trials of metformin. The commonest reasons for exclusion at the full text stage were the presence of an active comparator or the absence of text describing the collection of data for cardiovascular events as a study outcome or adverse event.

**Fig. 2:**
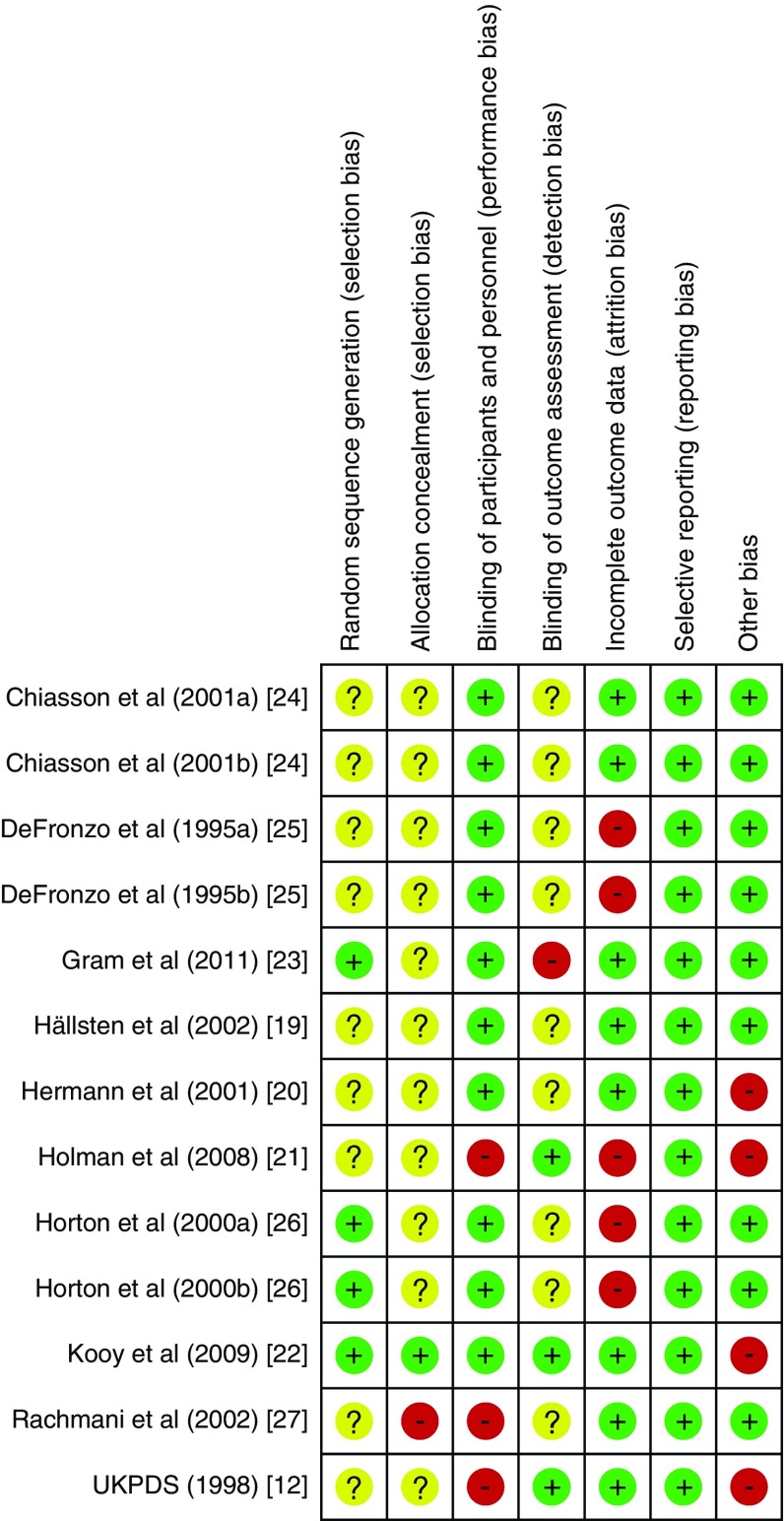
Risk of bias summary. Review authors’ judgements about each risk of bias item for included studies. Risk of bias was assessed according to the methods recommended by the Cochrane Collaboration. Question mark, unclear risk of bias; negative sign, high risk of bias; positive sign, low risk of bias

The characteristics of included studies are shown in Table 1. Studies reported between 1995 and 2011. Six were undertaken in Northern Europe [12, 19–23], six in North America [24–26] and one in Israel [27]. Three studies were open-label [12, 21, 27], one of which was a trial of cessation of metformin [27]. Of the ten placebo-controlled trials, six included other glucose-lowering drugs. We identified four trials including 417 patients allocated to metformin that simply compared metformin with placebo and collected data on cardiovascular outcomes [19, 24–26]. In total 2079 patients with type 2 diabetes were allocated to metformin, and a similar number to comparison groups, in the included studies. Duration of follow-up ranged from 6 to 212 months; three studies followed patients up for more than 4 years [12, 21, 22]. No studies were assessed as having low risk of bias (Fig. 2). The average age of recruited participants ranged from 53 to 65 years and exceeded 60 years in two studies [22, 27]. Trial participants tended to be mainly white, overweight/obese (average baseline BMI ranged from 28.7 to 34.2 kg/m^2^), with longstanding (average duration ranged from 0 years in two studies [19, 21] to 15 years [27]) and poorly controlled diabetes (average baseline HbA_1c_ was less than 8% [63.9 mmol/mol] in only four studies [12, 19, 21, 22] and ranged from 6.9% [51.9 mmol/mol] to 9.1% [76.0 mmol/mol]).

**Table 1:** Characteristics of included studies and study participants

Authors and year of study [reference]	Intervention group; control group	Met/control (*n*)	Inclusion criteria	Follow-up (months)	Age (years)	BMI (kg/m^2^)	Diabetes duration (years)	HbA_1c_ (%)	HbA_1c_ (mmol/mol)	Male (%)
Chiasson et al 2001a [24]	Met; Plac	83/83	Diet alone, HbA_1c_ 7.2–9.5% (55.2–80.3 mmol/mol)	36	57.9	30.7	7.5	8.2	66.1	73.5
Chiasson et al 2001b [24]	Met + Mig; Mig + Plac	76/82	Diet alone, HbA_1c_ 7.2–9.5% (55.2–80.3 mmol/mol)	36	58.9	29.5	6.1	8.3	67.2	77.6
DeFronzo et al 1995a [25]	Met; Plac	143/146	Obese, diet alone	7	53.0	29.9	6.0	8.4	68.3	43.4
DeFronzo et al 1995b [25]	Met + Glib; Glib + Plac	213/209	OW/obese, FBG > 7.8 mmol/l	7	55.0	29.0	7.8	8.8	72.7	46.0
Hällsten et al 2002 [19]	Met; Plac	13/14	New diagnosis or diet alone, FBG 6.1–11.0 mmol/l	6	57.8	29.9	0.0	6.9	51.9	61.5
Hermann et al 2001 [20]	Met + Ins; Plac + Ins	16/19	OW/obese, on Ins, HbA_1c_ > reference +2%^b^	12	56.9	33.6	13.0	9.1	76.0	43.8
Holman et al 2008 [21]	Met; diet	342/411	OW/obese, FBG > 6.1–15.0 mmol/l	212	53.0	31.6	0.0	7.3	56.3	45.9
Horton et al 2000a [26]	Met; Plac	178/172	HbA_1c_ 6.8–11% (50.8–96.7 mmol/mol)	6	56.8	29.6	4.5	8.4	68.3	68.0
Horton et al 2000b [26]	Met + Nat; Met + Plac	172/179	HbA_1c_ 6.8–11% (50.8–96.7 mmol/mol)	6	58.4	30.0	4.5	8.4	68.3	58.7
Kooy et al 2009 [22]	Met + Ins; Plac + Ins	196/194	On ins	51	64.0	30.0	14.0	7.9	62.8	41.3
Rachmani et al 2002 [27]	Continue Met; stop Met	195/198	Met, creatinine 132–220 μmol/l, HF, raised LFTs	48	65.0	28.7	15.0	8.6	70.5	52.8
UKPDS 1998 [12]	Met + Sul; Sul	268/269	FBG 6.1–15.0 mmol/l on Sul	79	59.0	29.7	7.1	7.5	58.5	57.2
Gram et al 2011 [23]^a^	Met +/− Asp Ins +/− Ros +/− NPH Ins; Plac +/− Asp Ins +/− Ros +/− NPH Ins	184/187	HbA_1c_ > 7.0% (53.0 mmol/mol), BMI > 25 kg/m^2^	24	56.0	34.2	8.5	8.6	70.5	64.1

^a^Factorial design

^b^An inclusion criterion of an HbA_1c_ value higher than the upper reference limit + 2% (21.8 mmol/mol)

Asp Ins, insulin aspart; FBG, fasting blood glucose; Glib, glibenclamide (known as glyburide in the USA and Canada); HF, heart failure (NYHA class 3–4); Ins, insulin; LFT, liver function test; Met, metformin; Mig, miglitol; Nat, nateglinide; OW, overweight; Plac, placebo; Ros, rosiglitazone; Sul, sulfonylurea

**Fig. 3:**
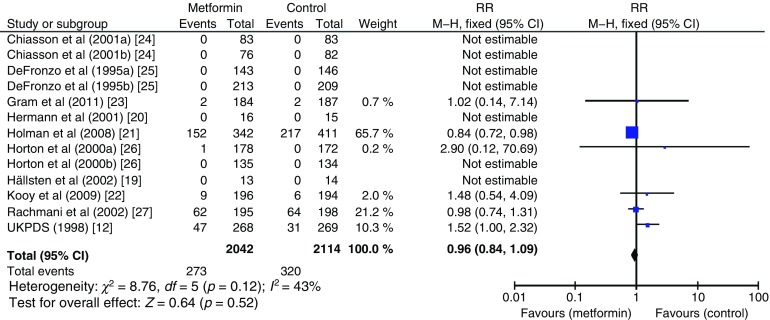
Forest plot showing the effect of metformin on risk of all-cause mortality

**Fig. 4:**
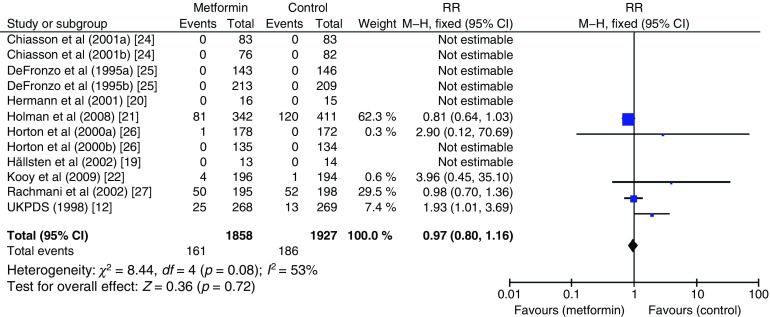
Forest plot showing the effect of metformin on risk of cardiovascular death

**Fig. 5:**
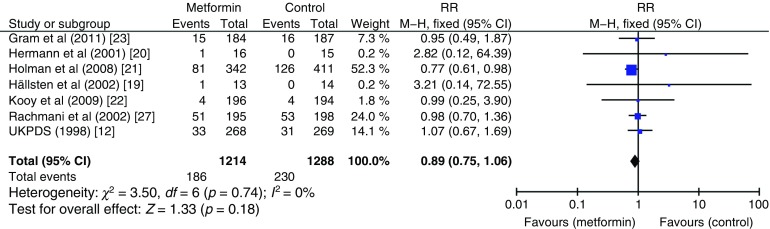
Forest plot showing the effect of metformin on risk of myocardial infarction

**Fig. 6:**
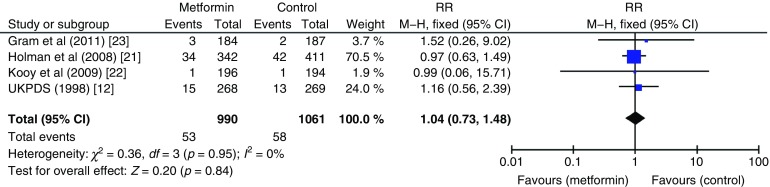
Forest plot showing the effect of metformin on risk of stroke

**Fig. 7:**
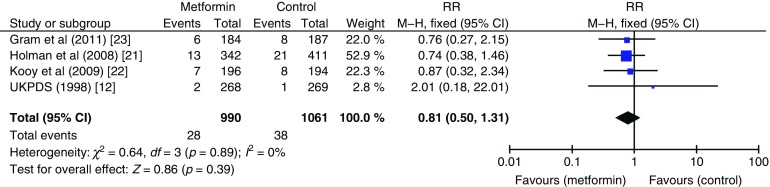
Forest plot showing the effect of metformin on risk of peripheral vascular disease

**Fig. 8:**
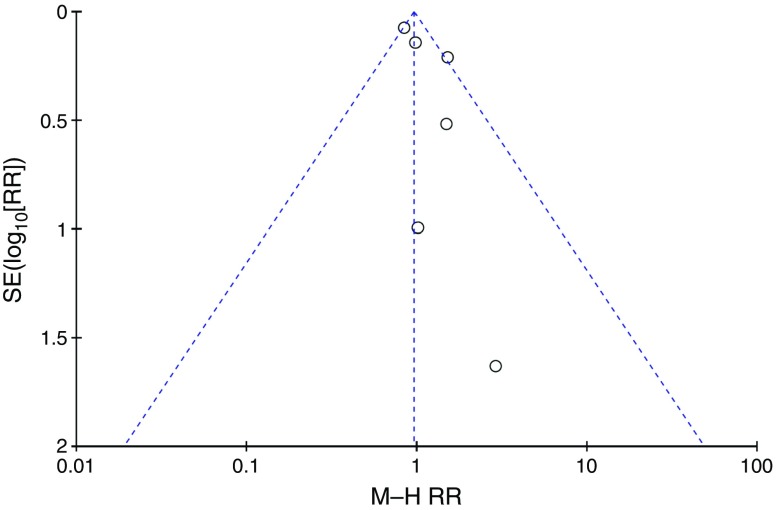
Funnel plot of effect size estimates for all-cause mortality to assess risk of publication bias. Circles represent M–H RR estimates for all-cause mortality comparing metformin vs control groups

The effect of metformin on risk of all-cause mortality (Fig. 3), cardiovascular death (Fig. 4), myocardial infarction (Fig. 5), stroke (Fig. 6) and peripheral vascular disease (Fig. 7) is shown. All outcomes, with the exception of risk of stroke, favoured metformin, with limited heterogeneity between studies, but none achieved statistical significance. Effect sizes (M–H RR) ranged from 0.81 (95% CI 0.50, 1.31) for peripheral vascular disease to 1.04 (95% CI 0.73, 1.48) for stroke. Summary estimates were based on a small number of events: 347 cardiovascular deaths in five studies, 416 myocardial infarcts/ischaemic heart disease events in seven studies and 111 strokes in four studies. One study, the UKPDS [21], contributed the majority of data to the summary estimates, with weights ranging from 52.3% for myocardial infarction to 70.5% for stroke. We undertook a sensitivity analysis replacing data from the longer term follow-up of UKPDS [21] with the original published data [12]. This led to small changes in the pooled estimates that more strongly favoured metformin for risk of stroke but more strongly favoured comparison groups for risk of myocardial infarction and peripheral vascular disease. All of the pooled estimates in the sensitivity analysis remained non-significant (metformin vs control). The funnel plot (Fig. 8) did not suggest that we had omitted trials demonstrating metformin-associated increased mortality.

## Discussion

In spite of its long history, we identified only 13 studies, including just over 2000 patients with type 2 diabetes allocated to metformin, that addressed our study question, and only four randomised-controlled cardiovascular endpoint trials simply comparing metformin with placebo among patients with type 2 diabetes. Metformin monotherapy appears safe and, while there is a suggestion of benefit, there remains uncertainty about whether it reduces risk of cardiovascular disease. According to our review it is possible that metformin reduces risk of all-cause mortality by up to 16% but it could increase risk of stroke by up to 48%. Metformin is the recommended first-line treatment worldwide for patients with type 2 diabetes. However, in contrast to some newer treatments, cardiovascular endpoint trial data for metformin are largely derived from small studies among relatively young, overweight/obese, North American and Northern European patients with poorly controlled diabetes. Metformin demonstrates cardiovascular safety as per the 2008 FDA guidance, but its use for prevention of cardiovascular disease among older individuals, those with HbA_1c_ less than 8% (63.9 mmol/mol), non-white ethnic groups and people living outside North America and Northern Europe is not well supported by trial evidence. Furthermore, while not specifically covered in this review, there remains concern about the observed increased risk of mortality associated with the addition of metformin to sulfonylurea treatment [14, 15].

The reports of all included trials either suggested the possibility of bias or provided insufficient information to allow risk of bias to be assessed. The one trial that appeared to exhibit low risk of bias for all but one criterion seemed to be compromised by clinically important baseline differences between study groups [22]. The majority of data for this review came from the UKPDS [21], a seminal trial concerning the effectiveness and safety of treatments for type 2 diabetes, albeit exhibiting a number of previously discussed limitations that might influence its interpretation [28]. These include the small size (only 342 patients were allocated to metformin), lack of placebo and double-blinding, ‘subgroup’ nature of the analysis with updated statistical significance thresholds, potential for between-group differences in management of other cardiovascular risk factors, unacceptably poor level of glycaemic control in the comparison group by current standards, and attrition over the near 18-year follow-up. Including data from the long-term follow-up of the UKPDS [21] introduces a number of assumptions that may lead to underestimation of effects, in particular the extent of any ‘legacy effect’ of treatment with metformin in the early part of the trial. However, a sensitivity analysis replacing the longer term follow-up with the original UKPDS trial data [12] increased the width of the 95% CIs but did not significantly change our findings.

### Strengths and weaknesses

We used a sensitive search strategy and systematically searched literature databases and reference lists of previous systematic reviews. However, we only searched three databases and may have excluded trials that are not indexed on MEDLINE or EMBASE or are unpublished. We only included trials that ‘isolated’ the effects of metformin in order to distinguish between benefits of metformin and harms associated with comparator drugs. With the exception of screening of some of the full text articles, the reviewing process was undertaken by two authors independently. We undertook quality assessment but included all trials meeting our pre-specified criteria. Definitions of the different cardiovascular outcomes varied between studies, particularly for peripheral vascular disease, which included angiographic findings in Kooy et al [22] but was restricted to amputation or death because of peripheral vascular disease in Holman et al [21]. We erred on the side of sensitivity to maximise the number of events, for example, including ‘ischaemic heart disease’ endpoints in Gram et al [23] as myocardial infarctions. Reporting of adverse events was inconsistent; consequently, for trials in which cardiovascular events were not specified as study outcomes, it was not always clear from the text of articles whether or not these data were collected as part of the monitoring for adverse events. It was also not possible to obtain clarification from authors and so we may have excluded some studies in which relevant data had been collected. However, the number of missed events is likely to be small in studies in which cardiovascular disease was not the main focus and, hence, the impact on summary estimates and conclusions negligible.

### Comparison with existing literature

Unsurprisingly, given the influence of the UKPDS data on this analysis and the overlap of the review question and included studies, our results are broadly similar to those of previous reviews published within the last 10 years [14, 15, 29]. In Selvin et al’s meta-analysis of trials of metformin vs any comparator, effect sizes for cardiovascular and all-cause mortality (M–H OR 0.74 [95% CI 0.62, 0.89] and M–H OR 0.81 [95% CI 0.60, 1.08], respectively) more strongly favoured metformin [29], mainly because of the inclusion of the open-label, active-comparator trial, the Comparative Outcomes Study of Metformin Intervention versus Conventional (COSMIC) Approach Study [30]. Lamanna et al’s meta-analysis, which included trials of diabetes prevention and trials with active comparators (in particular rosiglitazone), reported no overall effect of metformin on cardiovascular events (M–H OR 0.94 [95% CI 0.82, 1.07]) [15]. When analysis was restricted to comparisons of metformin with placebo or no drug treatment, metformin appeared to be beneficial (M–H OR 0.79 [95% CI 0.64, 0.98]). Our results most closely mirror those of Boussageon et al [14], who reported no effect of metformin on all-cause mortality (M–H RR 0.99 [95% CI 0.75, 1.31]) or cardiovascular mortality (M–H RR 1.05 [95% CI 0.67, 1.64]), but did not include the extended follow-up data from the UKPDS.

### Implications

Metformin lowers glucose and, hence, reduces symptoms of hyperglycaemia. It has a good safety profile, even among patients with impaired renal function [31], is relatively well tolerated and may even reduce cancer incidence and mortality [32], although this was not confirmed in a meta-analysis of trials [33]. The number, size, quality, reporting and findings of randomised trials of metformin have resulted in continuing uncertainty regarding whether it reduces risk of diabetes-related complications, particularly cardiovascular disease. Furthermore, there is a lack of cardiovascular endpoint data directly relevant to a significant proportion of the patients with type 2 diabetes worldwide for whom metformin is the recommended first-line medication. This contrasts with the evidence now available for newer and more expensive glucose-lowering drugs, such as empagliflozin [34] and liraglutide [10], and of course for medications targeting different risk factors, for example statins. Nevertheless, metformin is included on the WHO model list of essential medicines, a list of ‘the most efficacious, safe and cost-effective medicines for priority conditions’ and the ‘minimum medicine needs for a basic health-care system’. It is unlikely that patients, practitioners and ethics committees are all sufficiently close to equipoise to enable a large, double-blind, placebo-controlled, cardiovascular endpoint trial of metformin among patients with diabetes. It is also doubtful that a suitable industry, charity or government funder could be identified. While such a trial might reduce uncertainty about whether metformin is more effective than placebo, it would not inform common therapeutic dilemmas, such as which of the many available glucose-lowering drugs or combination of drugs to use, in which order to use them and for which patient. Possible, at least partial, solutions include the use of electronic health records to facilitate large, long-term, pragmatic, efficient trials comparing the effect of different treatments (new and old) on cardiovascular outcomes, plus the increased use of factorial trials in which an industry-sponsored new medication can be evaluated alongside older drugs such as metformin. There has been considerable hope and hype concerning the potential for precision medicine to inform treatment decisions [35], but progress is hampered by our lack of understanding about the mechanisms of action of metformin and a focus on intermediate endpoints. Publication of cardiovascular outcome data from adverse event reporting in trials of metformin would increase the data available for meta-analysis, thereby reducing the uncertainty of effect size estimates, but the small number of additional events are unlikely to lead to definitive conclusions.

Metformin reduced the incidence of diabetes by 31% among people with glucose levels just below the diagnostic threshold in the US Diabetes Prevention Programme [36], and by 26% in the Indian equivalent [37]. Although, these estimates were inflated because of participants undergoing outcome assessment while still taking metformin [38]. Proponents of a medical solution to what is essentially a societal problem are advocating the widespread use of metformin to ‘treat’ those at risk of diabetes. Indeed, metformin is now licensed in some countries for this indication. This effectively amounts to starting glucose-lowering treatment early in order to prevent the onset of diabetes and the need for glucose-lowering treatment, the aim of which is to reduce symptoms and risk of complications. Given that people at risk of diabetes do not have symptoms attributable to hyperglycaemia, the rationale for recommending metformin would be considerably strengthened if trial evidence was available demonstrating that the use of metformin in people at risk of diabetes reduced risk of complications, such as cardiovascular disease. Perhaps, therefore, there is greater interest, opportunity and need for a cardiovascular endpoint trial evaluation of metformin among people without diabetes than among those already living with the condition. While acknowledging that metformin has pleiotropic effects, if it was shown to be effective in such a trial, the near-linear relationship between HbA_1c_ and risk of cardiovascular disease and death [39], and the somewhat arbitrary diagnostic threshold for diabetes, might also reinforce the reputation of metformin for treating diabetes. Metformin is cheap, widely available, safe, backed by pharmaco-epidemiological and anecdotal evidence following up to 60 years of regular use in practice, and appears more likely to reduce risk of cardiovascular disease than increase it. Albeit, the latter assessment is based on a few small trials, with notable limitations, among an unrepresentative subset of patients. Newer agents that could potentially be used early in the course of the disease are now available, and are backed by data from recent rigorous cardiovascular endpoint trials. However, they remain very expensive and lack data on long-term use. Perhaps in spite of, rather than because of, the evidence, metformin is likely to remain the first-line treatment for the hyperglycaemia associated with type 2 diabetes for the foreseeable future.

## Electronic supplementary material

ESM Downloadable slideset (PPTX 1.57 mb)

## Abbreviations

FDA: Food and Drug Administration
M–H: Mantel–Haenszel
UKPDS: UK Prospective Diabetes Study
