# Supplementary material for: Impact of metformin on cardiovascular disease: a meta-analysis of randomised trials among people with type 2 diabetes
Source: Diabetologia. 2017 Aug 2;60(9):1620–9. doi: 10.1007/s00125-017-4337-9 (PMC5552849; doi:10.1007/s00125-017-4337-9)
Supplement: Supplementary file 1 — (PPTX 1.57 mb) [file 125_2017_4337_MOESM1_ESM.pptx]

## Slide 1
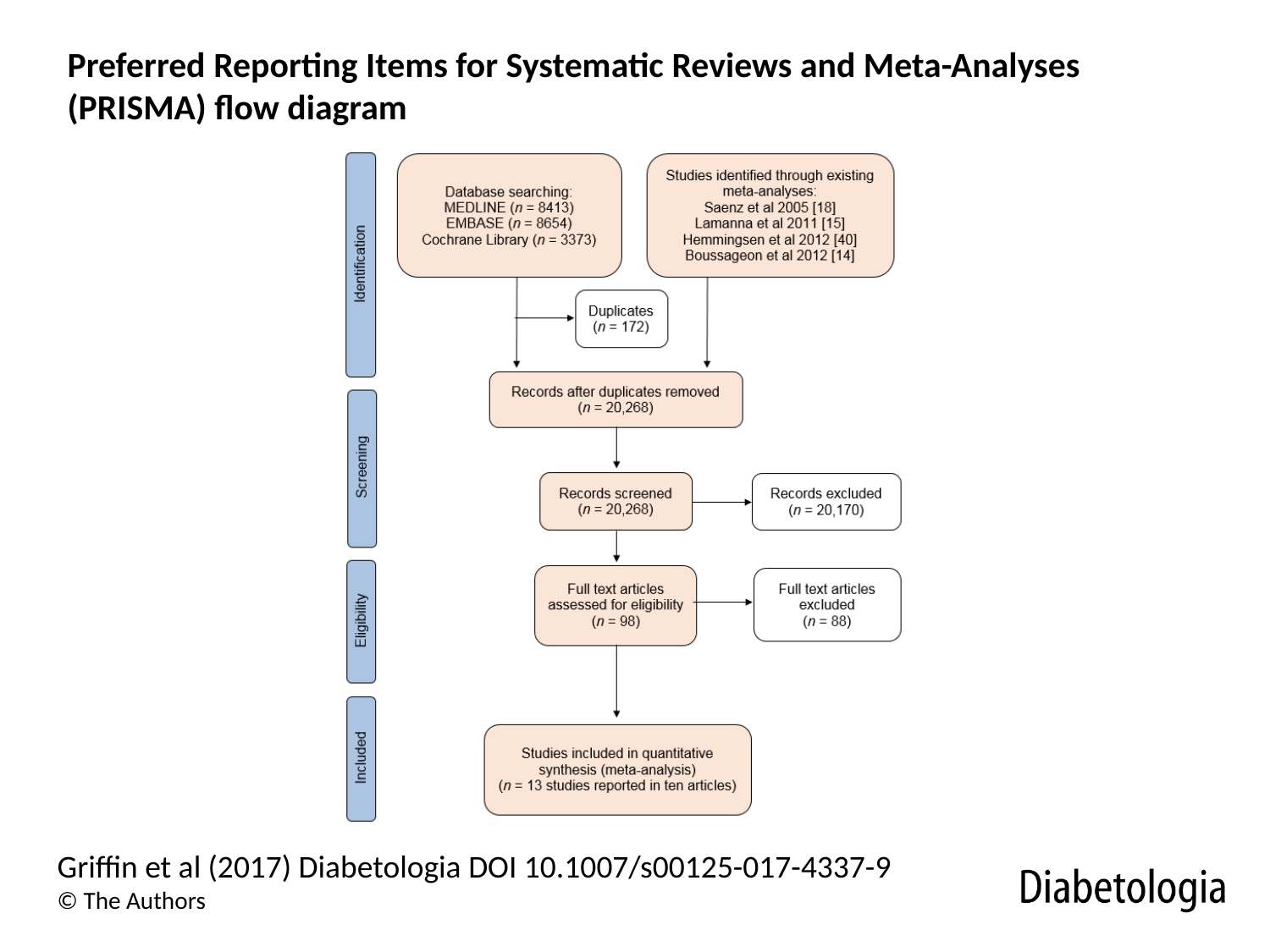

Preferred Reporting Items for Systematic Reviews and Meta-Analyses (PRISMA) flow diagram
Griffin et al (2017) Diabetologia DOI 10.1007/s00125-017-4337-9
© The Authors

## Slide 2
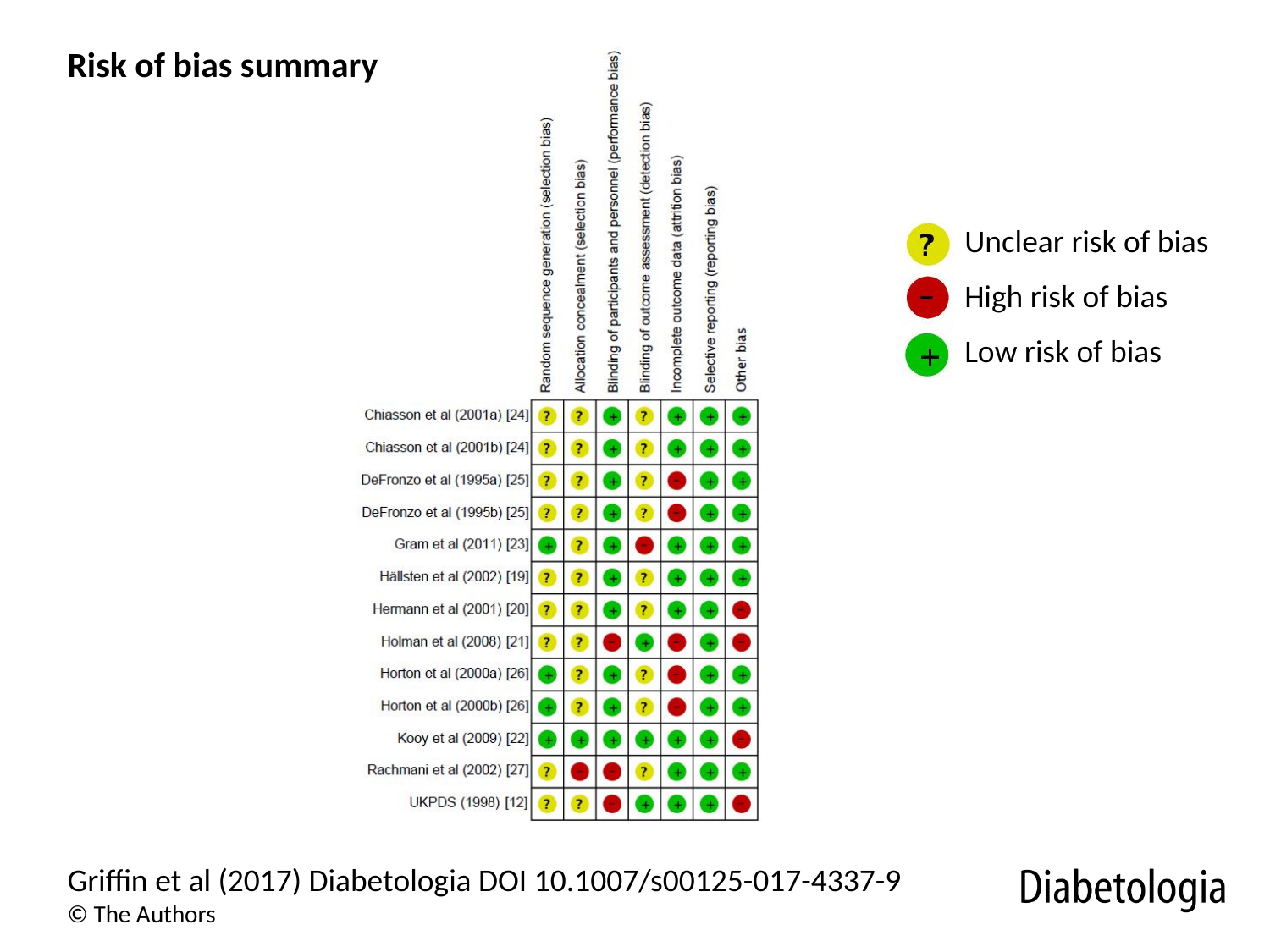

Risk of bias summary
Unclear risk of bias
High risk of bias
Low risk of bias
Griffin et al (2017) Diabetologia DOI 10.1007/s00125-017-4337-9
© The Authors

## Slide 3
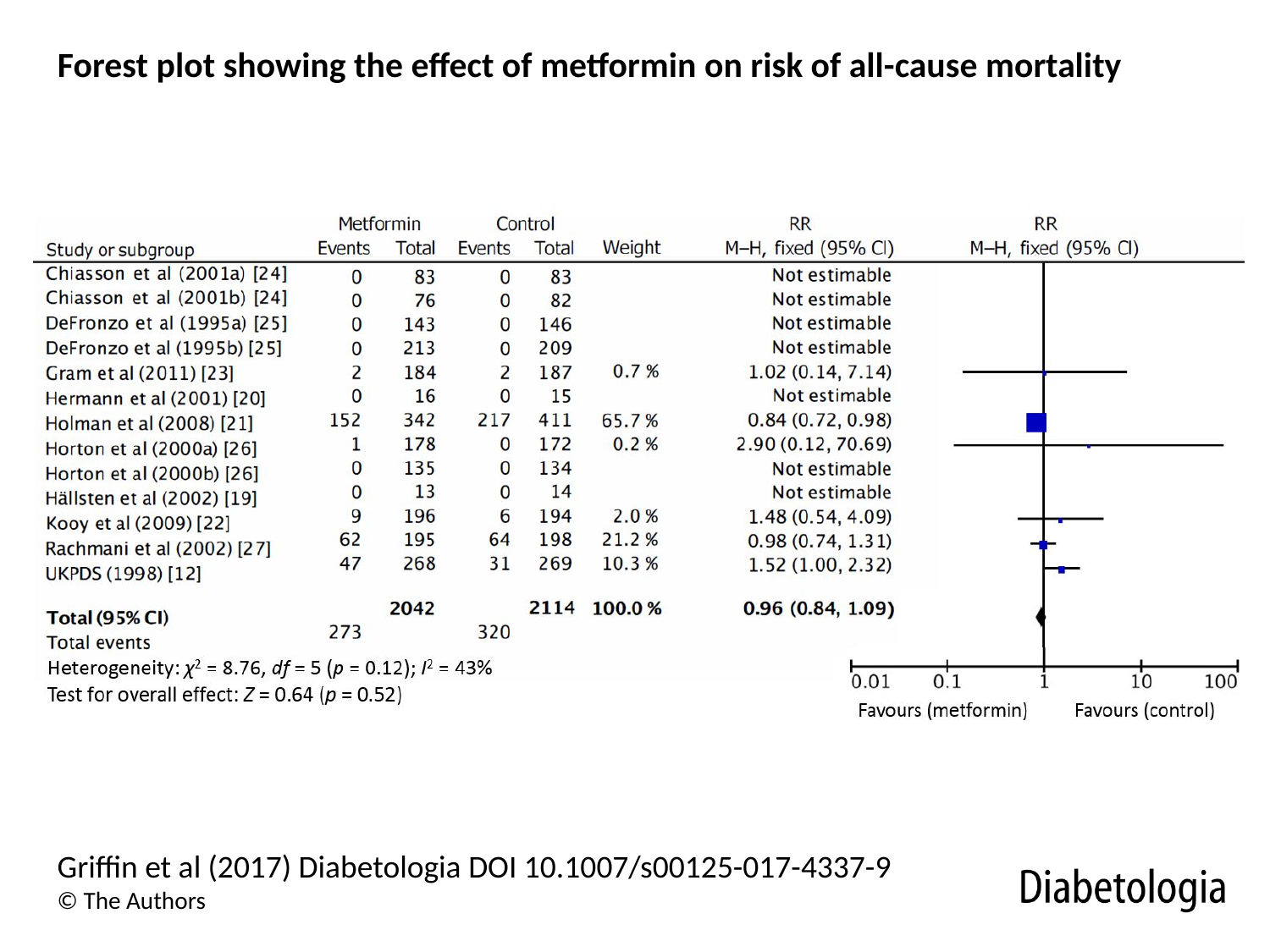

Forest plot showing the effect of metformin on risk of all-cause mortality
Griffin et al (2017) Diabetologia DOI 10.1007/s00125-017-4337-9
© The Authors

## Slide 4
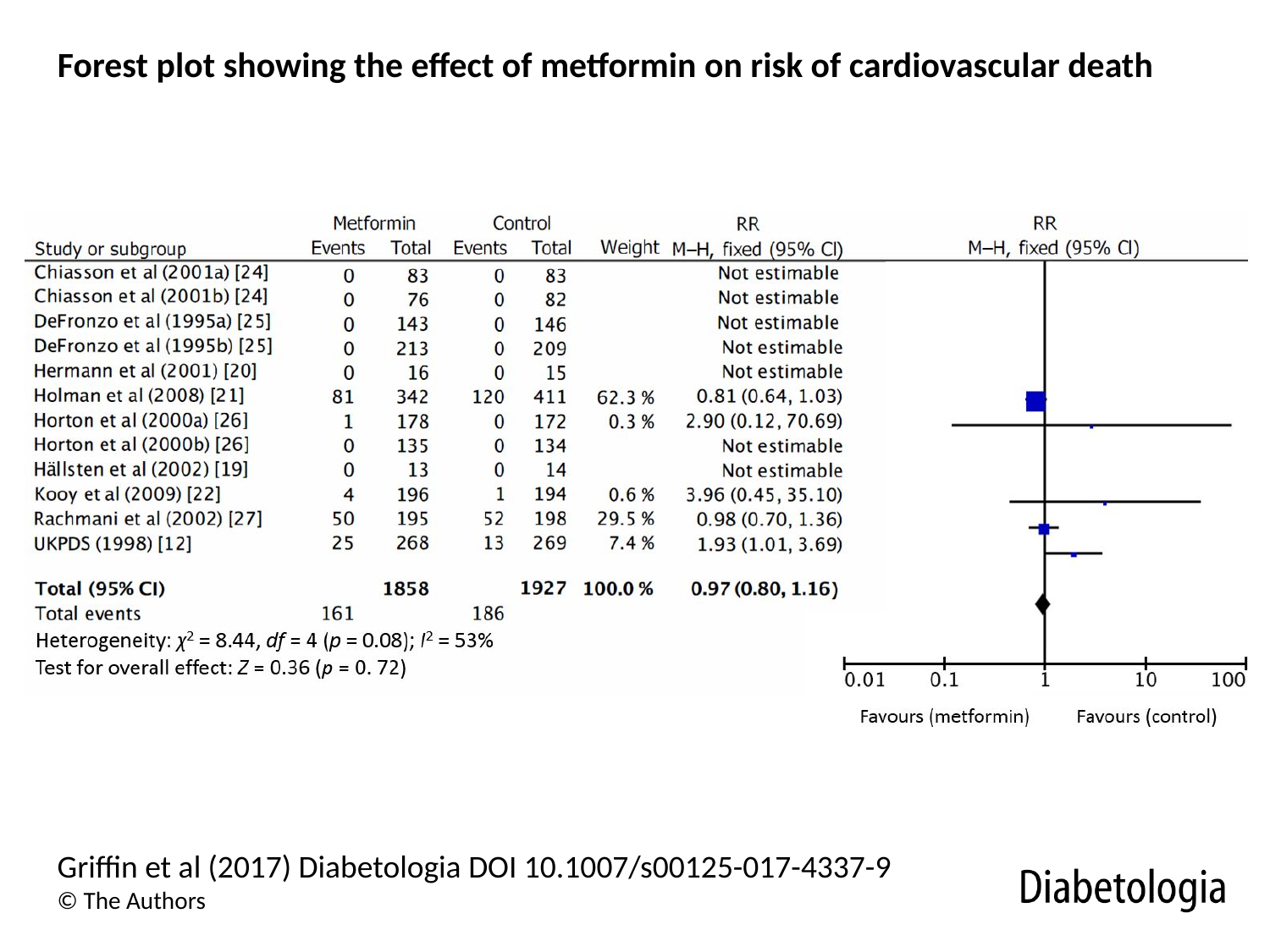

Forest plot showing the effect of metformin on risk of cardiovascular death
Griffin et al (2017) Diabetologia DOI 10.1007/s00125-017-4337-9
© The Authors

## Slide 5
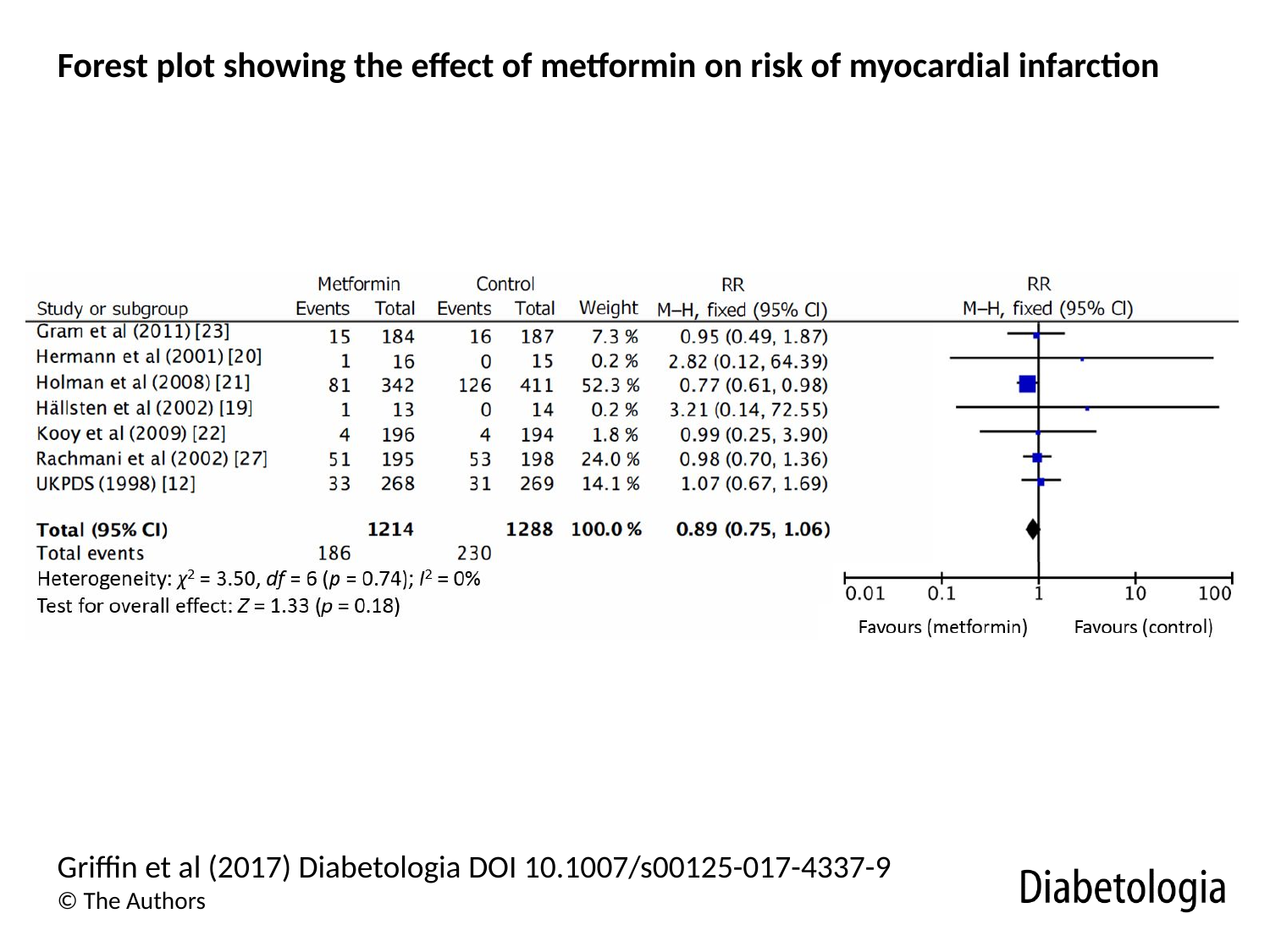

Forest plot showing the effect of metformin on risk of myocardial infarction
Griffin et al (2017) Diabetologia DOI 10.1007/s00125-017-4337-9
© The Authors

## Slide 6
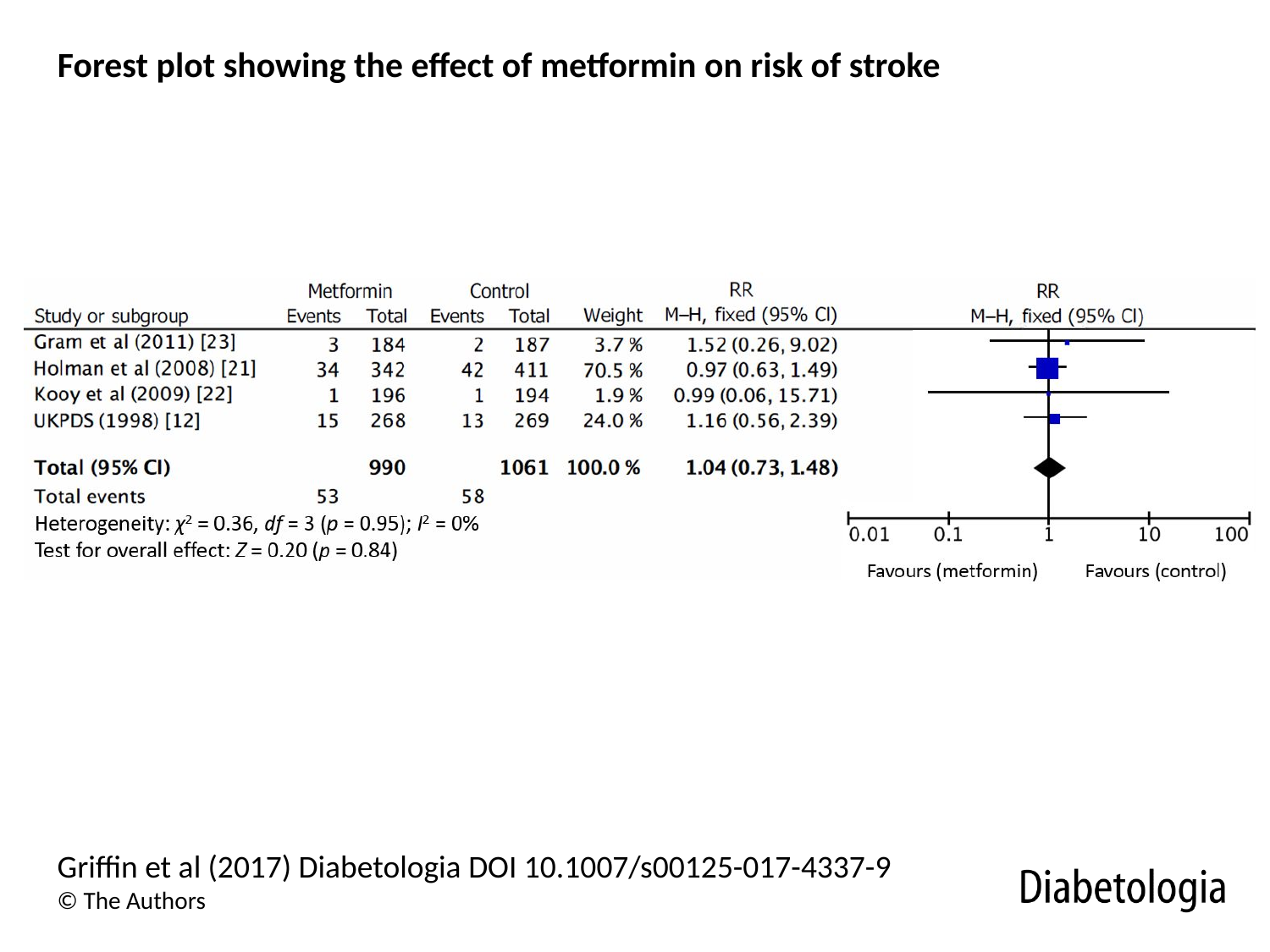

Forest plot showing the effect of metformin on risk of stroke
Griffin et al (2017) Diabetologia DOI 10.1007/s00125-017-4337-9
© The Authors

## Slide 7
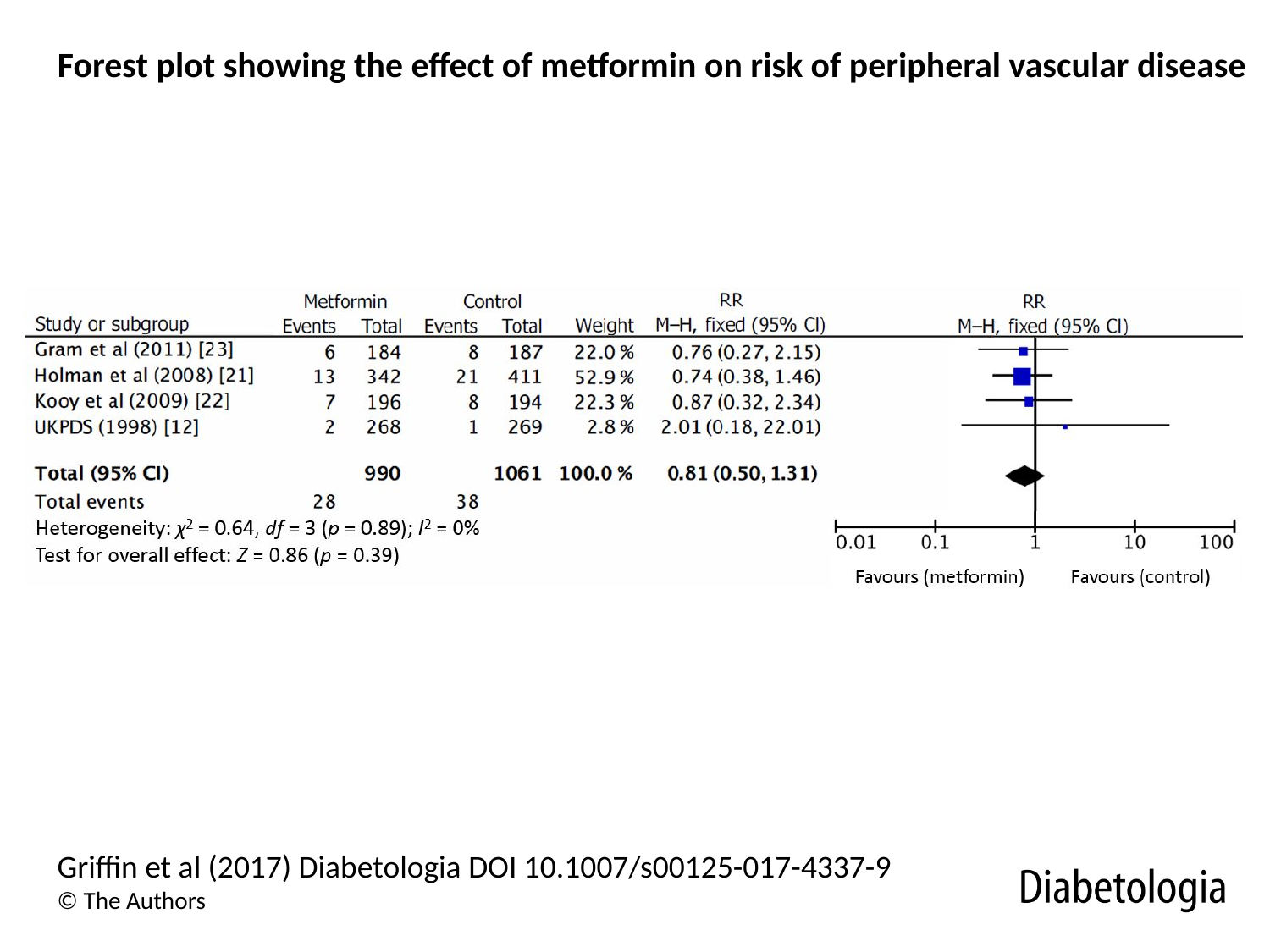

Forest plot showing the effect of metformin on risk of peripheral vascular disease
Griffin et al (2017) Diabetologia DOI 10.1007/s00125-017-4337-9
© The Authors

## Slide 8
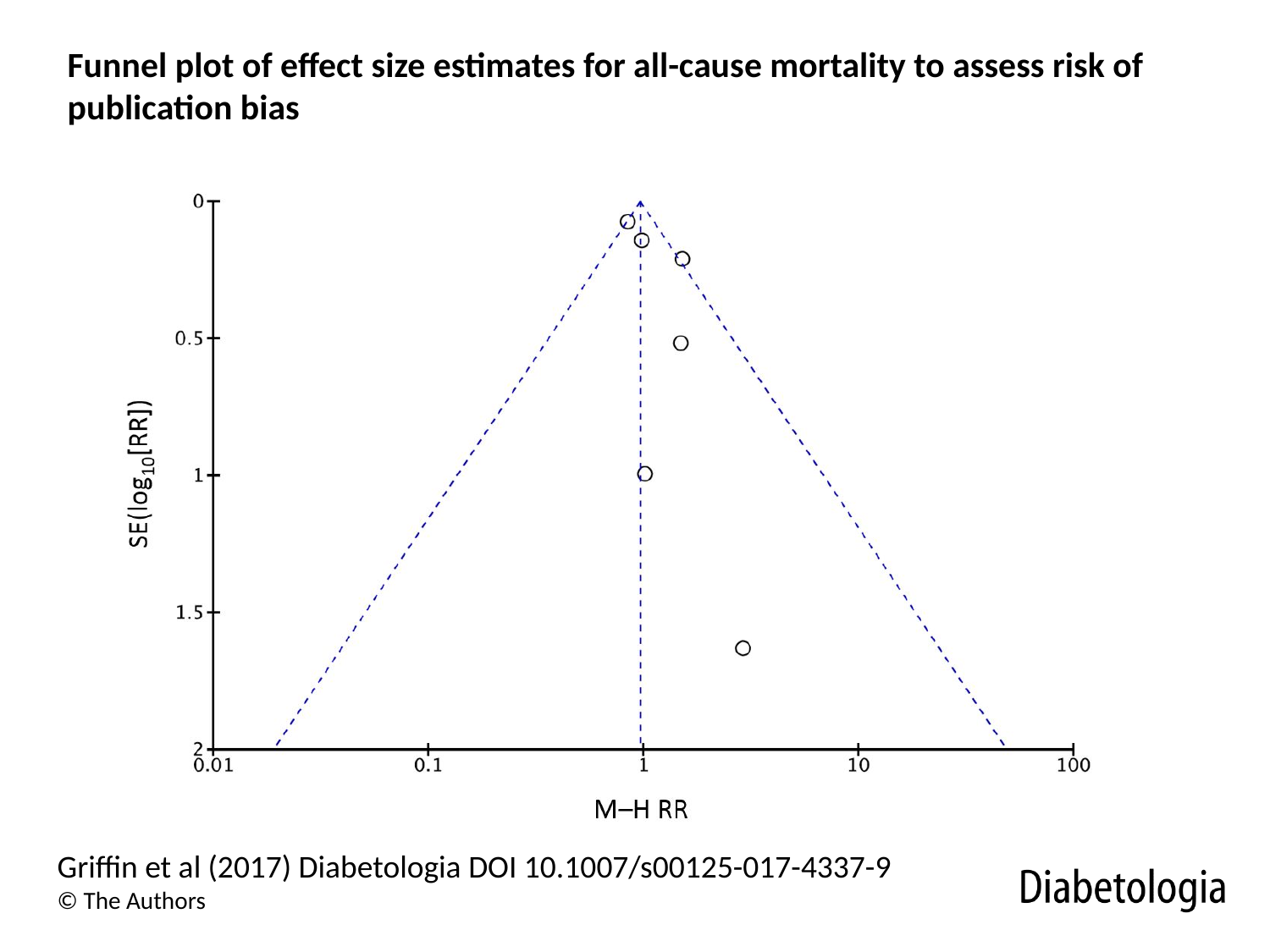

Funnel plot of effect size estimates for all-cause mortality to assess risk of publication bias
Griffin et al (2017) Diabetologia DOI 10.1007/s00125-017-4337-9
© The Authors
